# NPY1 Receptor Agonist Modulates Development of Depressive-Like Behavior and Gene Expression in Hypothalamus in SPS Rodent PTSD Model

**DOI:** 10.3389/fnins.2017.00203

**Published:** 2017-04-19

**Authors:** Lidia Serova, Hannah Mulhall, Esther Sabban

**Affiliations:** Department of Biochemistry and Molecular Biology, New York Medical CollegeValhalla, NY, USA

**Keywords:** stress, neuropeptide Y, corticotropin releasing factor, glucocorticoid receptor, FKBP5, depression, PTSD

## Abstract

Delivery of neuropeptide Y (NPY) to the brain by intranasal infusion soon after traumatic stress has shown therapeutic potential, and prevented development of many behavioral and neuroendocrine impairments in the single prolonged stress (SPS) animal model of PTSD. Therefore, we examined whether the Y1R preferring agonist [Leu^31^Pro^34^]NPY is sufficient to prevent development of SPS induced depressive-like behavioral changes, and hypothalamic gene expression as obtained with intranasal NPY intervention. Male Sprague-Dawely rats were given intranasal infusion of either NPY (150 μg/rat), a low (68 μg /rat), or high (132 μg/rat) dose of [Leu^31^Pro^34^]NPY or vehicle immediately following the last SPS stressor, left undisturbed for 1 week and then tested for depressive-like behavior together with naïve unstressed controls. Vehicle treated animals had elevated immobility forced swim test (FST) and reduced sucrose preference, which were not observed in animals given NPY or the higher dose of [Leu^31^Pro^34^]NPY. This dose of [Leu^31^Pro^34^]NPY, like NPY, also prevented the SPS-elicited induction of CRF mRNA in the mediobasal hypothalamus. However, [Leu^31^Pro^34^]NPY did not prevent, but rather enhanced, the SPS-triggered induction of GR and FKBP5 mRNA levels in the mediobasal hypothalamus. Thus, [Leu^31^Pro^34^]NPY may be as effective as NPY and displays therapeutic potential for preventing development of depressive-like behaviors and dysregulation of the CRF/HPA system in PTSD. However, due to its different effects compared to NPY on GR and FKBP5 a broader agonist, such as NPY, may be more desirable.

## Introduction

Considerable evidence indicates that in the CNS neuropeptide Y (NPY) can attenuate the response to stress, and has therapeutic potential for PTSD as well as for depression (reviewed in Heilig, 2004; Morales-Medina et al., 2010; Wu et al., 2011; Sah and Geracioti, 2013; Enman et al., 2015; Reichmann and Holzer, 2016; Sabban et al., 2016; Schmeltzer et al., 2016). Our recent studies revealed proof of concept that delivery of NPY to the brain shortly before or immediately after exposure to traumatic stress can prevent the development of many PTSD associated behavioral and neuroendocrine impairments. Intranasal infusion of NPY after exposure to traumatic stress in the single prolonged stress (SPS) protocol, rodent model of PTSD, averted the elevation of anxiety, depressive-like behavior and hyperarousal observed in vehicle treated animals a week or more afterwards. Intervention with intranasal NPY also provided resistance against prolonged activation of the hypothalamic pituitary adrenal (HPA) axis, and molecular changes in multiple brain regions, including the mediobasal hypothalamus (Serova et al., 2013; Laukova et al., 2014; Sabban et al., 2015a, 2016).

At least four NPY receptors, Y1R, Y2R, Y4R, and Y5R, mediate the biological effects of NPY (Michel et al., 1998; Hirsch and Zukowska, 2012). The Y6 subtype is truncated, non-functional in humans and absent in the rat. The NPY receptors associate with Gi/Go and regulate several signaling cascades leading to hyperpolarization by inhibiting calcium channels and activation of GIRK or I_H_ channels, inactivation of adenylyl cyclase and thus cAMP dependent pathways and mobilization of intracellular calcium by phospholipase C and phosphatidyl inositol kinase. NPY can lead to changes ERK or CREB signaling resulting in alterations in gene expression (reviewed in Brothers and Wahlestedt, 2010; Sah and Geracioti, 2013).

Y1R, Y2R, and Y5R are abundantly expressed in brain areas implicated in anxiety and depression (reviewed in Kask et al., 2002; Heilig, 2004; Eva et al., 2006). The importance of Y1 transmission in depressive disorders was emphasized in the Flinders Sensitive Line, a genetic model of depression. In these animals, hippocampal and hypothalamic Y1 receptor mRNA levels were lower and Y1 receptor binding higher than in the control Flinders Resistant Line (Jiménez-Vasquez et al., 2007). The beneficial effect of NPY was also observed in the acute model of depression, likely mediated by the Y1 receptors (Redrobe et al., 2002; Goyal et al., 2009). Moreover, antidepressant like effects of agmatine occur via the NPYergic system and probably by stimulation of the Y1 receptor subtype (Kotagale et al., 2013). Immunohistochemistry showed that neuroendocrine CRF neurons in the PVN coexpress Y1R. Direct infusion of the Y1 preferring agonist [Leu^31^Pro^34^]NPY into the PVN increased c-Fos and phosphorylated CREB expression in populations of CRF/Y1r-ir cells and elevated plasma corticosterone levels (Dimitrov et al., 2007). This suggests that NPY afferents and subsequent activation of NPY Y1 receptors play an important role in the regulation of the HPA.

Due to the widespread distribution of NPY into the brain following intranasal infusion (Sabban et al., 2016), it remains to be determined the activation of what receptor subtypes are sufficient for NPY's stress-reductive and therapeutic effects. In addition, a more selective agonist than NPY may able to be effective at low dose, and provide less opportunity for potential side effects.

In this study we examined whether the Y1 receptor preferring agonist [Leu^31^Pro^34^]NPY is able to provide selective protective effects on traumatic stress triggered depressive-like behaviors and changes in hypothalamic gene expression.

## Materials and methods

### Materials

[Leu^31^, Pro^34^]-Neuropeptide Y (human, rat) was purchased from Tocris. NPY was synthesized by NeoScientific (Cambridge, MA). They were stored lyophilized at −80°C and dissolved in distilled water immediately before infusion.

### Animals

All experiments were performed in accordance with the National Institute of Health Guide for the Care and Use of Laboratory Animals and approved by Institutional Animal Care and Use Committee at NYMC and the USAMRMC Animal Care and Use Review Office. Male Sprague-Dawley rats (150–160 g) were purchased from Charles River (Wilmington, MA) and housed (4 per cage) in a barrier area on 12 h light/dark cycle at 23 ± 2°C with *ad libitum* access to food and water.

### Experimental design

**Figure 1:**
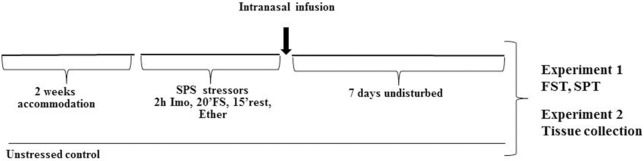
**Experimental design**. After 2 weeks accommodation, rats were exposed to SPS stressor consisting of 2 h immobilization stress (Imo) followed immediately by 20 min forced swim (FS), and after 15 min rest exposed to ether vapors until loss of consciousness. While still under the influence of ether they received intranasal infusion of either NPY, [Leu^31^Pro^34^]NPY, or vehicle. Control animals were unstressed. Seven days later they were either tested for depressive like behavior on the FST and SPT (Experiment 1) or euthanized and appropriate tissues collected (Experiment 2).

The experimental design is shown in Figure 1. After 2 week acclimation period, the rats were randomly assigned to the experimental or control groups (10 rats per group). SPS was performed between 9 a.m. and 2 p.m. as previously described (Serova et al., 2013). First, rats were subjected to a 2 h immobilization on metal board by taping the limbs with a surgical tape and restricting the motion of the head. Immediately afterwards, they were subjected to a 20 min forced swim in a plexiglass cylinder (50 cm height, 24 cm diameter, Stoelting, Wood Dale, IL) filled to two-thirds with 24°C fresh water. The animals were dried and allowed to recuperate for 15 min and then exposed to ether vapor until loss of consciousness.

While still under the influence of ether from the last SPS stressor, each rat received intranasal infusion of either: (1) 150 μg NPY; (2) 68 μg [Leu^31^Pro^34^]NPY; (3) 132 μg [Leu^31^Pro^34^]NPY, or (4) vehicle (distilled water). The infusion was administered, 10 μl into each nare, with pipetteman and disposable plastic tip. Extreme care was taken to avoid contact with the intranasal mucosa. Following administration, the head of the animal was held in a tilted back position for approximately 15 s.

Following the SPS procedure, animals left undisturbed (2 per cage) for 7 d and were then tested on the forced swim test and sucrose preference test (Experiment 1) or euthanized to determine changes in gene expression in the mediobasal hypothalamus (Experiment 2).

#### Forced swim test (FST)

Rats were examined in a modified version of the Porsolt swim test (Cryan et al., 2002) as previously described (Serova et al., 2013). They were put into the same plexiglass cylinders filled to two-thirds with 24°C fresh water for 5 min and their behavior was recorded. A trained individual blinded to the experimental group scored the time spent swimming, defined as movement of the forelimbs and hind limbs and the time spent immobile when the animal showed no movement, or only movements needed to keep its head above the water.

#### Sucrose preference test (SPT)

For the sucrose preference test (Briones and Woods, 2013), rats were trained to a two-bottle choice of drinking water and 1% sucrose solution for 2 days followed by 2 days of testing. On the day of testing, two pre-weighted bottles of 5% sucrose solution and tap water were presented. To prevent possible effects of side preference in drinking behavior, the position of the bottles was switched after 24 h of training or testing. No food or water deprivation was applied before or during the test. Liquid consumption from each bottle corrected by body weight was used to calculate sucrose solution intake, water intake and total consumption by the end of the 48-h period. Sucrose preference was calculated using the following equation: sucrose preference (%) = sucrose intake/(sucrose intake + water intake) × 100.

#### Gene expression in mediobasal hypothalamus

A week after the SPS stressors, the rats were sacrificed and the mediobasal hypothalamus containing paraventricular nucleus (PVN) without the arcuate nucleus was isolated and immediately frozen in liquid nitrogen at kept at −80°C. Total RNA was isolated with RNeasy Plus Mini Kit (Qiagen, Valencia, ML). This kit has been designed to isolate total RNA from animal tissues and obtain optimal RNA yield and purity. It also allows eliminating contamination by genomic DNA using gDNA eliminator columns. Briefly, the frozen samples were homogenized in lysate buffer containing β-mercaptoethanol with Polytron PT 1200E (Kinematica AG, Switzerland). After centrifugation the supernatant was transfered and centrifuged through gDNA eliminator spin columns. After addition of 70% ethanol, RNA was precipitated on the RNeasy spin columns, washed and eluted with RNase-free water. RNA concentration was evaluated by sepectrophotometry (NanoDrop 2000, Thermo Fisher Scientific, Pittsburgh, PA). The ratio of absorbance at 260 to 280 nm was about 2.0. Overall average yield of isolated total RNA was 5–8 μg per 10 mg of brain tissue which is within in the best range provided by Qiagen's protocol.

The relative levels of CRF, GR, and FKBP5 mRNAs were determined by RT-qPCR. Reverse transcription of 1,000 ng of RNA was performed with the RevertAid First Strand cDNA Synthesis kit (Thermo Fisher Scientific, Hanover Park, IL) using an oligo dT primer at 42°C for 60 min in MyCycler (BioRad, Hercules, CA). For qPCR, the cDNA (33.2 ng in 2 μl) was mixed with 12.5 μl of FastStart Universal SYBR Green Master Rox (Roche Diagnostics, Indianapolis, IN) and 1 μl of the following primer sets: CRF (Crh, NM_031019.1, cat. no. PPR44803B, Qiagen); glucocorticoid receptor (GR) (Nt3c1, NM_012576.2, cat no. PRR52805B, Qiagen); FKBP5 (Fkbp5, NM_001012174.1, cat. no. PPR51629B, Qiagen) and glyceraldehyde-3-phosphate dehydrogenase (GAPDH; Gapdh, NM_017008.4, forward 5′-TGGACCACCCAGCCCAGCAAG-3′, reverse 5′-GGCCCCTCCTGTTGTTATGGGGT-3′), to a final volume 25 μl in PCR-96-Microplate (Axygen Scientific, Union City, CA). The primers for CRF, GR and FKBP5 were validated experimentally by Qiagen to amplify a single amplicon (125, 81, 96 bp respectively) with uniform PCR efficiency. The amplicon for GAPDH (140 bp) was shown to be proportional to RNA input. PCR was performed on ABI7900HT Real-Time PCR instrument (Applied Biosystems, Carlsbad, CA). The data were analyzed with SDS Software 2.4 (Applied Biosystems). The melting curves were examined to verify a single amplicon at the expected melting temperature. Ct values were in the range of 27–29 for CRF, 25–28 for GR and FKBP5, and 16–17 for GAPDH. Data were normalized to GAPDH mRNA (not altered by experimental conditions) and expressed as the relative fold changes calculated using the ΔΔCt method (Livak and Schmittgen, 2001).

### Statistical analysis

Data were analyzed using Prizm 4 (GraphPad) software. Following confirming normality with D'agatino and Pearson Omnibus Normality Test, and data were analyzed by one way ANOVA followed by Tukey's Multiple Comparison Test for differences among the groups. Values of *p* ≤ 0.05 were considered significant.

## Results

### Intranasal administration of [Leu^31^Pro^34^]NPY prevented the SPS elicited depressive-like behavior

**Figure 2:**
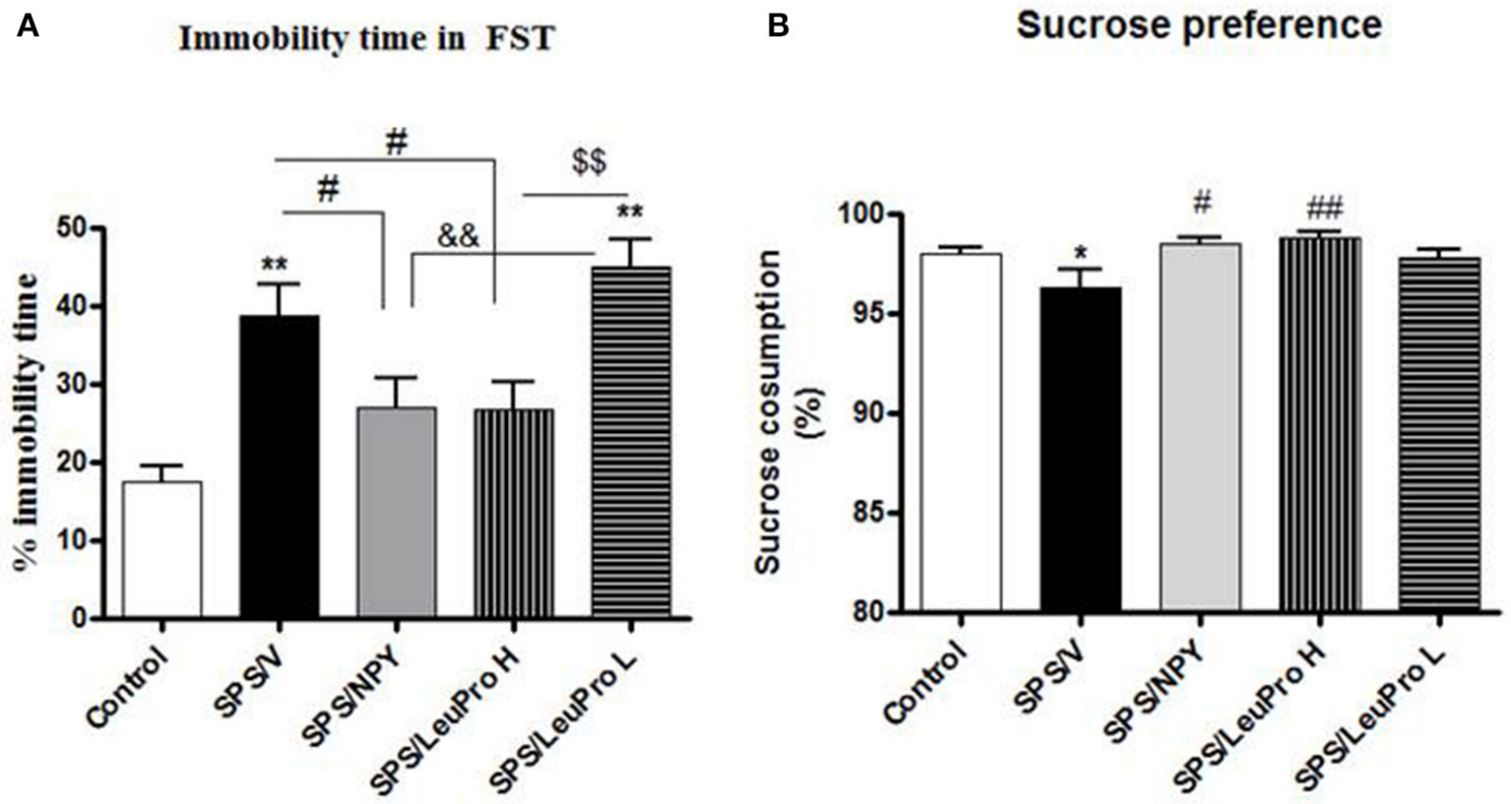
**Depressive-like behavior**. Immediately after the last SPS stressor rats were infused with vehicle (SPS/V), 150 μg NPY (SPS/NPY), 132 μg [Leu^31^Pro^34^]NPY (SPS/LeuPro H), or 64 μg [Leu^31^Pro^34^]NPY (SPS/LeuPro L) or unstressed (Controls). Testing for depressive-like behavior began 7 days later. **(A)** Immobility time on FST. **(B)** Sucrose preference. Data are presented as mean ± SEM. *n* = 8–10 per group. ^*^p < 0.05, ^**^p < 0.01 compared to Controls; ^#^*p* < 0.05, ^##^*p* < 0.01 compared to SPS/V; ^&&^*p* < 0.01 compared to SPS/NPY; ^$$^*p* < 0.01 compared to SPS/LeuPro H.

Initially, we examined the ability of two doses of the Y1R preferring agonist [Leu^31^Pro^34^]NPY to change despair or depressive-like behavior in forced swim test (FST) of rats subjected to SPS stressors (Figure 2). Animals were given intranasal infusion of either: NPY (150 μg/rat), low (68 μg/rat) or high (132 μg/rat) dose of [Leu^31^Pro^34^]NPY, or vehicle immediately following the last stressor (ether) of the SPS protocol. They were left undisturbed for 1 week and then tested on the FST (Figure 2A) together with naïve unstressed controls. One way ANOVA showed a significant impact of treatment on time immobile (*F* = 9.5, *p* < 0.0001). Rats that received vehicle (SPS/V) spent longer time immobile compared to the unstressed controls (*p* < 0.01). Administration of NPY, as previously observed (Serova et al., 2013), prevented development of this despair behavior. Despite similar body weight in all the SPS treated groups, the SPS/NPY group of rats spent less time immobile compared to the SPS/V group (*p* < 0.05) and did not differ from the unstressed controls. The results with [Leu^31^Pro^34^]NPY depended on the dose used. The low dose was not effective to change SPS-induced immobility time in FST (*p* > 0.05, SPS/LeuPro L vs. SPS/V). However, the immobility time with the higher dose was different than with the low dose (*p* < 0.01) and significantly reduced compared to the vehicle treated group (*p* < 0.05, SPS/LeuPro H vs. SPS/V). Similarly, treatment with NPY as well as the higher, but not the lower, dose of [Leu^31^Pro^34^]NPY prevented the reduction in sucrose reference observed in the vehicle treated group (Figure 2B). Therefore, only the higher dose was used in the further experiments.

### Effects of NPY and [Leu^31^Pro^34^]NPY on single prolonged stress (SPS) elicited molecular changes in the mediobasal hypothalamus

**Figure 3:**
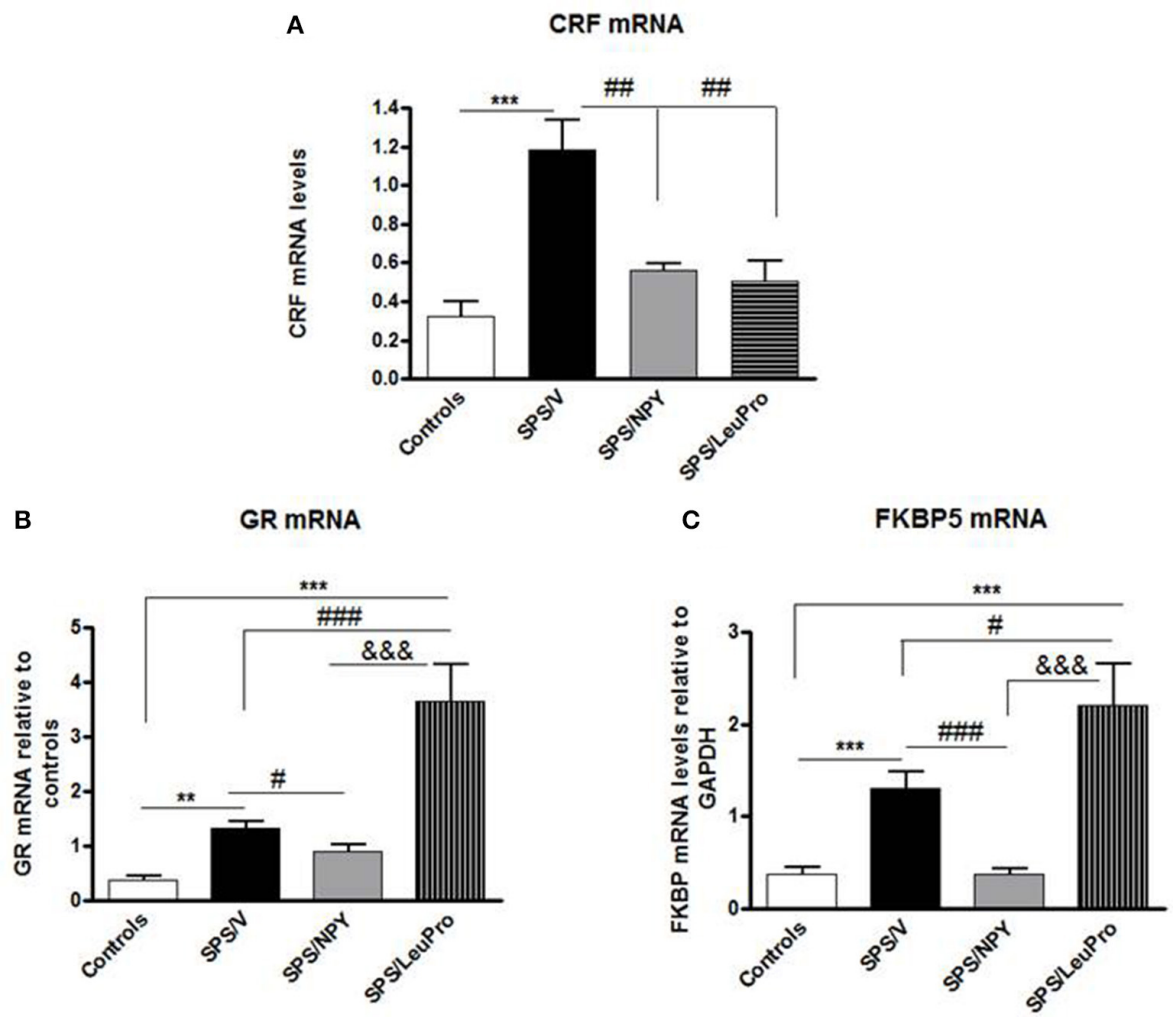
**Changes in mRNA levels for several genes in the mediobasal hypothalamus**. Data are presented as mean ± SEM. *n* = 8–10 per group. **(A)** CRF mRNA levels. ^***^p < 0.001 compared to controls; ^##^*p* < 0.01 compared to SPS/V. **(B)** GR mRNA levels. ^**^p < 0.01, ^***^p < 0.001 compared to controls; ^#^*p* < 0.05, ^###^*p* < 0.001 compared to SPS/V; ^&&&^*p* < 0.001 compared to SPS/LeuPro. **(C)** FKBP5 mRNA levels. ^***^p < 0.001 compared to Controls; ^#^*p* < 0.05 ^###^*p* < 0.001 compared to SPS/V; ^&&&^*p* < 0.001 compared to SPS/LeuPro.

In the next experiment, we examined the effect of early intervention with intranasal NPY, or [Leu^31^Pro^34^]NPY on SPS-elicited changes in expression of several genes in the mediobasal hypothalamus, an integrative center in the regulation of HPA axis (Figure 3). One way ANOVA revealed significant differences in CRF mRNA levels among animals with different treatments (*F* = 10.0, *p* < 0.0001, Figure 3A). Tukey's multiple comparison test showed that [Leu^31^Pro^34^]NPY was similar to NPY in preventing the elevation of CRF mRNA. An induction of CRF mRNA was observed only in the vehicle treated group (*p* < 0.01 compared to controls, SPS/NPY or SPS/LeuPro).

Since the GR plays a crucial role in the negative feedback regulation of HPA axis we examined GR mRNA levels. ANOVA showed significant impact of treatment (*F* = 15.0, *p* < 0.0001, Figure 3B). While, significantly increased by SPS in vehicle infused animals compared to controls (*p* < 0.01) this did not occur in rats given NPY infusion. They had GR mRNA levels similar to the controls and decreased compared to the SPS/V group (*p* < 0.01). Surprisingly, in animals with [Leu^31^Pro^34^]NPY infusion, GR mRNA levels were significantly higher than in controls, SPS/V or SPS/NPY groups (*p* < 0.001).

The levels of FKBP5 mRNA were also affected by treatment (*F* = 10.0, *p* < 0.0001, Figure 3C). In agreement with our previous published results (Laukova et al., 2014), similar to CRF and GR, mRNA levels of FKBP5 are also elevated by SPS in the mediobasal hypothalamus. The pattern of changes in FKBP5 gene expression was analogous to those of GR mRNA levels. There was elevation of gene expression of FKBP5 by SPS as shown in vehicle treated group but not in animals given NPY (*p* > 0.01 vs. controls or SPS/NPY). However, rats administered [Leu^31^Pro^34^]NPY had a greater induction of FKBP5 mRNA levels, which was higher than in any of the other groups.

## Discussion

The results of this study suggest an important role for the Y1R in protection from development of the depression-like symptoms following the exposure to severe stress. Infused immediately after the application of SPS stressors, the higher dose of [Leu^31^Pro^34^]NPY was able to prevent development of SPS-induced increase in immobility time in FST. This dose was also protective against long lasting elevation of CRF mRNA levels in the mediobasal hypothalamus. However, the Y1R preferring agonist did not prevent the SPS elicited effects on gene expression of GR and FKBP5 in this brain region, which were even higher than in SPS group treated with vehicle.

The findings demonstrated that [Leu^31^Pro^34^]NPY, when given at a similar dose as NPY, is equally effective to ameliorate the development of SPS-elicited depressive-like behavior as shown by both the FST and sucrose preference test. The importance of the Y1R on basal activity on FST has previously been observed. Intracerebral infusion of the Y1R agonist [Leu^31^Pro^34^]PYY, or NPY, 30 min before the test significantly increased time mice spent swimming compared to controls (Redrobe et al., 2002). Administration of [Leu^31^Pro^34^]NPY in olfactory bulbectomized rat model of depression reduced depressive related features in open field test (Goyal et al., 2009). Moreover, Y1R deficient mice (−/−) displayed greater immobility time in FST than the +/+ wild type controls (Karlsson et al., 2008) confirming the importance of the Y1R. Our data revealed for the first time that stimulation of Y1R immediately after exposure rats to SPS stressors provides a long-lasting anti-depressive-like effect. It remains to be determined whether Y1R agonists are sufficient to also produce the reduction in SPS triggered symptoms of anxiety obtained with intranasal NPY.

The results also suggest that beneficial effects of intranasally infused NPY in SPS-induced despair or depressive-like behavior is mediated by its activation of Y1 receptors. Although [Leu^31^Pro^34^]NPY is assumed to be specific for the Y1 receptor we cannot rule out a contribution of the Y5 receptor subtype. It has been shown that [Leu^31^Pro^34^]NPY also has efficacy at the Y5R (Gerald et al., 1996). The rat hypothalamus also expresses Y5R to some extent. In rat hypothalamic homogenates approximately 20% of specific binding fit the pharmacological profile of Y5 receptors (Widdowson et al., 1997). It is also have been found that endogenous NPY acts via PVN Y1 and Y5 receptors to change sympathetic nerve activation and heart rate (Cassaglia et al., 2014). Thus the Y1R preferring agonist, [Leu^31^Pro^34^]NPY given in relatively high dose could also interact with Y5 receptors. Therefore, our data with [Leu^31^Pro^34^]NPY may represent additive activation of both receptors. Further studies with more doses of [Leu^31^Pro^34^]NPY and different agonists will help determine the lowest effective dose and distinguish their selective roles.

[Leu^31^Pro^34^]NPY was sufficient to prevent the SPS elicited rise of CRF gene expression. CRF plays a key role in integrating neural, endocrine, and behavioral responses to stressful stimuli. During stress, CRF initiates the activation of the HPA axis. Released from the hypothalamus, CRF stimulates ACTH synthesis and release from the anterior pituitary. This evokes glucocorticoid secretion from the adrenal cortex into circulation. Glucocorticoids, via GR, mediate many of their physiological responses to stress. Within the hypothalamus, GR plays a crucial role in direct glucocorticoid feedback by repressing CRF biosynthesis and release and thus enabling appropriate termination of the stress response.

Elevated expression of CRF in the mediobasal hypothalamus has been linked to a depressive-like state. Many antidepressant drugs have delayed onset of clinical efficacy and in rats, long-term administration of clinically effective antidepressant drugs resulted in reduction in CRF mRNA expression levels in the hypothalamic PVN (Brady et al., 1991). The over-expression of CRF in the PVN appears to be a common neuroendocrine abnormality for depressive states in animals (Mironova et al., 2013). Depression and PTSD are frequently is co-morbid. Patients with PTSD and animal models of PTSD display dysregulations of the HPA axis on several levels, such as blood glucocorticoid and ACTH concentration, expression of GR receptors in many brain regions as well as GR receptor modulator glucocorticoid sensitive co-chaperone FK506-binding protein 5 (FKBP5) (Yehuda, 2009; Mehta et al., 2011; Knox et al., 2012; Laukova et al., 2014). We previously observed that SPS has a long lasting effect on activation of the HPA axis. A week after exposure to SPS stressors, corticosterone and ACTH in plasma and CRF, GR, and FKBP5 mRNAs in the mediobasal hypothalamus were still significantly above levels in unstressed animals (Laukova et al., 2014; Sabban et al., 2015b).

The results of experiments presented here revealed than activation of Y1 receptors with [Leu^31^Pro^34^]NPY immediately after exposure to SPS stressors can prevent development of abnormal expression of CRF in the PVN which might also related to physiologically normal immobility time in the FST in these rats. In contrast to rats treated with NPY, rats infused with [Leu^31^Pro^34^]NPY still had robustly elevated mRNA levels for GR and FKBP5. Moreover the levels of these two mRNAs were even higher than in the rats administered with vehicle. A dissociation of the effects on CRF gene expression from those on GR and FKBP5 in the mediobasal hypothalamus was also observed with the melocortin 4 receptor antagonist, HS014 (Serova et al., 2014).

Although hypothalamic GR plays a major role in glucocorticoid-dependent feedback mechanism regulating CRF gene expression, our results suggest that normalization of CRF gene expression with [Leu^31^Pro^34^]NPY may be mediated by other pathways. The PVN receives inputs from the medial amygdala indirectly via the bed nucleus of the stria terminalis and from the ventral hippocampus via interneurons (López et al., 1999; Ulrich-Lai and Herman, 2009) which indirectly control CRF expression. In this regard, disruption of GR in the PVN led to HPA hyperactivity but did not affect anxiety and despair-like behavior (Laryea et al., 2013). In contrast to CRF, GR synthesis in the mediobasal hypothalamus is not restricted to the PVN (Aronsson et al., 1988). Thus, our findings with GR may reflect stimulation of Y1 receptors on different mediobasal hypothalamic cell types other than the PVN.

Overall the results indicate that while [Leu^31^Pro^34^]NPY can be similarly effective as NPY with therapeutic potential in PTSD for preventing development of depressive-like behaviors and dysregulation of CRF/HPA system at the level of the PVN. However, issues relating to its effect on GR and FKBP5 gene expression indicate that it might not be as useful therapeutically as NPY.

## Author contributions

Each of the authors made a significant contribution to the manuscript. ES and LS, design, analysis, interpretation of the data and writing the manuscript. HM, performance of experiment on gene expression, participation in interpretation of data, and in review of manuscript.

### Conflict of interest statement

The authors declare that the research was conducted in the absence of any commercial or financial relationships that could be construed as a potential conflict of interest.
